# Molecular Characterization of *Streptococcus agalactiae* Isolates from Pregnant Women in Kathmandu City

**DOI:** 10.1155/2020/4046703

**Published:** 2020-08-28

**Authors:** Kusum Shrestha, Anil Kumar Sah, Neetu Singh, Pramila Parajuli, Rameshwar Adhikari

**Affiliations:** ^1^Department of Microbiology, St. Xavier's College, Maitighar, Kathmandu, Nepal; ^2^Research Center for Applied Science and Technology (RECAST), Tribhuvan University, Kritipur, Kathmandu, Nepal; ^3^Annapurna Neurological Institute and Allied Sciences, Annapurna Research Center, Maitighar, Kathmandu, Nepal; ^4^Manmohan Memorial Medical College and Teaching Hospital, Kathmandu, Nepal

## Abstract

**Introduction:**

Group B streptococci (GBS) are globally recognized as one of the leading causes of neonatal sepsis and meningitis and is also known to cause adverse pregnancy outcomes such as stillbirths and miscarriages. Thus, detailed investigation of GBS in pregnant women has special significance in public health related researches.

**Objectives:**

The present study is aimed at evaluating the prevalence of GBS colonization among pregnant women in Kathmandu city.

**Methods:**

The study was carried out among 125 pregnant women at their trimester (35–37 weeks) (during the time period between January and June in 2018). The prevalence was determined by the culture method in HiCrome Strep B Selective Agar Base and then by using the PCR technique. The serotypes were evaluated by multiplex PCR analysis, while the antibiotics susceptibility tests were performed using the disk diffusion method.

**Results:**

Among 125 samples studied, GBS were recorded in 24 samples (implying a prevalence of 19.2%). Furthermore, using the multiplex PCR, among 24 GBS-positive samples, 13 (54.17%) were found to be typeable while 11 (45.83%) were nontypeable. The most abundant serotype recorded in this study was type III (33.33) while the serotypes IV, V, VI, VII, and VIII were not found.

**Conclusion:**

The isolates were sensitive towards some antibiotics such as linezolid and ceftriaxone 100%, whereas penicillin 50% and vancomycin 75% but were resistant to tetracycline and ertapenem. Serotype III was found to be predominant in the samples collected during the study period. The observed prevalence was significantly associated with the gestational period, whereas no relationship was found for other risk factors.

## 1. Introduction

*Streptococcus agalactiae* is the leading bacteria for causing early neonatal morbidity and mortality [1]. It is globally well known as one of the leading causes of neonatal sepsis and meningitis and also is known to cause adverse pregnancy outcomes such as stillbirth and miscarriages [2]. It is a Gram-positive, *β*-hemolytic opportunistic bacterium [3] that is found to colonize the genital and gastrointestinal tract of 10–40% of healthy women. Maternal colonization with GBS is the major risk factor for the development of invasive neonatal disease [4] and also is an important cause of infections to elderly individuals and immune-compromised patients [5]. Based on the capsular polysaccharide (CPS) antigens, GBS are classified into 13 serotypes. Among 13 variants, 9 variants, Ia, Ib, II, III, IV, V, VI, VII, and VIII, are considered as clinically important [6]. The capsular polysaccharides of GBS isolates are the important virulence factors with antiphagocytic purposes and therefore are used as the key components of developing new multivalent GBS vaccines [4]. The CPS has been used for serotype identification [7]. The GBS are distributed differently among age, parity, socioeconomic status, geographic region, sexual behavior, and so on, which may change over time period [8].

The Centers for Disease Control and Prevention (CDC) recommends penicillin and ampicillin as the first-line antibiotics for intrapartum prophylaxis in pregnant women who are carriers of GBS. In case of penicillin-allergic women, erythromycin and clindamycin [9] have been considered as alternative antibiotics because of their narrow spectrum of activity and ability to achieve high intra-amniotic concentrations [10].

The aim of the present study is to determine serotypes of clinical GBS isolates obtained from pregnant women population using the multiplex PCR assay in Kathmandu to obtain accurate information about the distribution of GBS serotypes in this region, which can provide some knowledge about the related infections.

## 2. Materials and Methods

### 2.1. Study Design

A hospital based prospective cross-sectional study was conducted to determine the rate of vaginal colonization among pregnant women and their antibiotics susceptibility pattern attending Manmohan Memorial Medical College and Teaching Hospital and Annapurna Neurological Institute and Allied Sciences, Kathmandu. Two swabbed samples were collected from each pregnant woman, one for culture and the other for Gram staining. A structured questionnaire was prepared to collect demographic data.

### 2.2. Inclusion Criteria

The pregnant women at their third trimester (from week 35 to week 37) visiting the aforementioned hospitals were included in this study.

### 2.3. Exclusion Criteria

Pregnant women who had received antibiotics within one week, had elective caesarean section, and refused to enroll in the present study were excluded from this study.

### 2.4. Study Variables

GBS Atr gene which comprises “CAA CGA TTC TCT CAG CTT TGT TAA” as forward primer and “TAA GAA ATC TCT TGT GCG GAT TTC” as reverse primer, respectively, serves as dependent variables.

Age, body mass, occupation, religion, formal education level, number of children, existence of chronic medical problems, neonatal death, and so on have been considered as independent variables.

### 2.5. Collection and Identification of Isolates

A total of 125 low vaginal swab samples of pregnant women were screened for the period between January and June 2018. The specimens were transferred to Amie's transport medium and processed following standard laboratory protocol. Direct plating was carried out by inoculating onto HiChrom Strep B Selective agar. The culture plates were hence incubated at 37°C for 36 hrs under anaerobic conditions. Conventional phenotypic methods including Gram staining as well as beta-hemolysis, catalase, oxidase, bacitracin resistivity test, and urease tests were used for presumptive identification of the isolates. For Gram staining, the test sample was flooded with crystal violet for 10–30 seconds followed by washing with distilled water. Gram's iodine was flooded for 60 seconds and washed with distilled water. The slide was flooded with alcohol acetone decolorizer for 10 seconds to remove excess of stains. Finally, safranin was used as counter stain for 30 seconds and washed with distilled water. The slide was allowed to air dry and was examined under the oil immersion microscopy [11].

### 2.6. Antimicrobial Susceptibility Test

*In vitro* antibiotic sensitivity test was performed by the modified Kirby Bauer disc diffusion method using MHA with 5% blood as recommended by Clinical and Laboratory Standard Institute (CLSI) guideline 2017. The antibiotics hence used were penicillin, erythromycin, chloramphenicol, tetracycline, vancomycin, ertapenem, linezolid, levofloxacin, clindamycin, and ceftriaxone. The details are included in the supplementary table (available here).

### 2.7. DNA Extraction

About 1–5 overnight isolated colonies of GBS were taken in a sterile microcentrifuge tubes; then 567 *μ*L of TE buffer, 30 *μ*L 10% SDS, and 5 *μ*L proteinase K were added and mixed thoroughly by vortexing and followed by incubation at 37°C for 1 hour. Afterwards, 100 *μ*l of 5 M NaCl and 80 *μ*L of buffer solution (containing 10% CTAB in 4 M NaCl solution) were added and mixed immediately, followed by incubation at 65°C for 10 minutes. Then, 0.4 mL of chloroform: isoamyl alcohol (24 : 1) was added, mixed thoroughly, and centrifuged for 4–5 minutes at 16,128 x g. The aqueous liquid phase was carefully transfer to clean microcentrifuge tube (should not be viscous). Then, 0.4 mL of phenol : chloroform : isoamyl alcohol (volume ratio 25 : 24 : 1) were added and mixed well, followed by a spin at 16,128 x g. The supernatant portion as separated was transferred to clean microcentrifuge tube and 600 *μ*L of isopropanol hence was added to get a precipitate then centrifuged at 16,128 x g for 5 min. The sample was incubated at −20°C for 2 hrs to overnight, followed by centrifuged for 15 min at 4°C, 16,128 x g. Then, the precipitate was washed with 70% ethanol and centrifuged for 5 min at 16,128 x g, discarded the supernatant, and allowed pellet to dry at room temperature and resuspended in 50 *μ*L of TE buffer. Then, it was stored at −20°C until further use.

### 2.8. Confirmation of Identified Isolates as GBS Using the PCR Assay

*S. agalactiae* specific 780 bp Atr gene (GenBank accession number: AF15135) were used as internal positive control for the PCR assay to confirm the identified isolates. The forward and reverse primer sequences CAA CGA TTC TCT CAG CTT TGT TAA and TAA GAA ATC TCT TGT GCG GAT TTC were used, respectively. The PCR reaction volume was 25 *μ*L including 1 *μ*L of bacterial DNA, 0.5 *μ*L of forward primer, 0.5 *μ*L of reverse primer, 12.5 *μ*L of 2x Taq Premix-Master mix, and 10 *μ*L of sterile double distilled water. Amplification of thermal cycles were as follows: an initial denaturation step for 5 min at 94°C, followed by 35 cycles of 94°C for 30 s, 55°C for 55 s, and 72°C for 1 min, and a final extension cycle of 72°C for 10 min using [12] Proflex, Thermofisher, USA. PCR products and 100-bp DNA size marker were run simultaneously on 1.5% agarose gel stained with DNA safe stain at 60 V for 1 hour. Finally, the agarose gel was visualized and photographed using UV Tech Cambridge.

### 2.9. Molecular Serotyping of GBS Isolates the Using Multiplex PCR Assay

Each isolate confirmed as GBS was examined for genotyping (molecular serotyping) using the multiplex PCR assays targeting nine cps genes introduced [7]. For this purpose, two reaction mixtures were prepared: (i) the mixture comprising the primers for Ia, Ib, II, III, and IV and (ii) the mixture containing the primers for V, VI, VII, and VIII (see in Table 1). The first reaction mixture contained 1 *μ*L of bacterial DNA, 0.5 *μ*L of each forward primer, 0.5 *μ*L of each reverse primer, 12.5 *μ*L of 2x Taq Premix-Master mix, and 6.5 *μ*L of sterile double distilled water. The second reaction mixture also contained the additional constituents same as for the first reaction mixture. The final volume of each reaction mixture was 25 *μ*L. Amplification of thermal cycles was as follows: an initial denaturation step for 3 min at 94°C, followed by 30 cycles of 94°C for 30 s, 58°C for 1 min, and 72°C for 1 min, and a final extension cycle of 72°C for 5 min. As mentioned in the PCR assay, PCR products were run on agarose gel and then photographed using UV trans-illuminator. The 100-bp DNA size marker was used in these assays [12].

The primers used in this study were GBS capsular gene cps1aH, cps2K, cps4N, cps5O, cps6I, cps7M, and cps8J for serotypes Ia, II, IV, V, VI, VII, and VIII, respectively, as well as cps1bJ and cpsIbK for the serotypes Ib-F and Ib-R, respectively. For serotype III, dltS genes were used following the standard procedures [7].

### 2.10. Data Collection Processing and Analysis

A questionnaire sheet used in this study was specifically developed and detailed interview was conducted to collect personal data of each participant considered in the study, detailing her demographic characteristics, and family history. Chi-square test was used to determine the association of independent variables. A value of *α* ≤ 0.05 has been assumed wherever applicable and 95% confidence intervals along with the exact *p* values have been presented. Values of *p* < 0.05 were considered statistically significant.

## 3. Results

The present study has shown that, among 125 tested vaginal swabs, 24 (19.2%) were positive for GBS. Among the positive, 13 (54.17%) were found typeable, whereas 11 (45.83%) were nontypeable. There was significant association found between GBS and gestational period whereas no association was determined for other risk factors.

**Figure 1:**
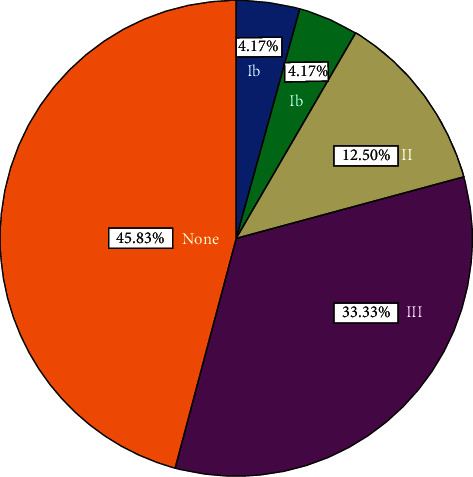
Percentage distribution of GBS serotypes (Ia, Ib, II, and III) of *S. agalactiae* based on cps genes as indicated.

Multiplex PCR detection was performed to differentiate among the nine capsular serotypes of *S. agalactiae* (Ia, Ib, and II–VIII).  Figure 1 presents the distribution of GBS serotype (Ia, Ib, II, and III) of *S. agalactiae* based on the respective cps genes. It can be found that serotype III was the predominant one (33.33%), followed by II (12.5%), Ia (4.17%), and Ib (4.17%), while serotypes IV–VIII were not detected. The overall prevalence for GBS colonization in different countries is reported to be 5–40% implying the regional variation of the world [13]. The reason for this observation is not clear so far. However, in developing countries like Nepal, the problem has not been adequately studied, and, as a result, the reports are hardly available in literature and government archives.

**Figure 2:**
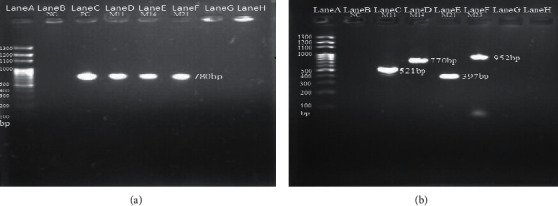
Photograph showing the results of gel electrophoresis from (a) the confirmatory PCR assay targeting 780 bp atr gene (lane A: 100 bp DNA size marker; lane B: negative control (NC); lane C: positive control (PC); lanes D to F: clinical GBS samples M11, M14, and M21) and (b) the multiplex PCR assay for genotyping of GBS (see the text for amplicons size-serotype correlations).

As mentioned earlier, the identified isolates of the GBSs were confirmed using the PCR assay, while the molecular serotyping was assessed by multiplex PCR tools. The results are presented in Figure 2. The results of gel electrophoresis are presented in Figure 2(a), which contain 100 bp DNA size marker in lane A, negative control in lane B, and positive control in lane C. Lanes D, E, and F comprise the clinical GBS samples M11, M14, and M21, respectively, as confirmatory specimens for GBS positive targeting 780 bp atr gene using the PCR assay.

Figure 2(b) shows the results of gel electrophoresis of the multiplex PCR assay for serotyping of confirmatory GBS (positive from the conventional PCR assay in Figure 2(a)), in which 100 bp DNA size marker and negative control found in lanes A and B, respectively, are the same as for lanes C, *D*, E, and F containing confirmatory GBS-positive samples M11, M14, M21, and M23, respectively, for serotyping. The amplicons of sizes 521 bp, 770 bp, 397 bp, and 952 bp denoted type Ia, type Ib, type II, and type dltS serotypes, respectively.

The expected PCR patterns were obtained with all primer pairs and serotypes, except for primers III-F and III-R. Due to their high degree of sequence similarity with these loci, type III strains were expected to cross-react with type Ia and II strains. Therefore, dltS gene representing serotype III was used with primer pair dltS-F and dltS-R targeting the GBS-specific dltS gene including it as an internal positive control [7].

## 4. Discussion

In this study, the rate of vaginal colonization among pregnant women was found to be 19.2%. Similar studies performed in several industrialized nations have shown different carriage rates: Canada 19.5% [14], the UK (Oxford) 21.3% [15], the USA 15–40% [16], and Sweden 25.3% [17]. The few studies published from developing countries have shown comparatively lower prevalence rates: Japan 16%, Korea 8%, Myanmar 7.1%, and Philippines 7.5% [18], and the exception in this trend was Zimbabwe, where colonization rates of 60.3% were reported [19]. The risk of a neonate to be colonized at birth is directly related to the intensity of maternal colonization [20]. The rate of vaginal colonization among pregnant women was found to depend on various risk factors, culture methods, type of medium used, time of pregnancy, race, origin, age, parity, and socioeconomic level [21].

Diet, climate, maternal hygiene, and culture methods, including the number and type of sites cultured and medium used, have accounted for some of these variations in the GBS colonization rates [22].

From Figure 1, it is illustrated that serotype III (33.33%) is found to be the most prevalent one followed by II (12.5%), Ia, and Ib (4.17%) and serotypes IV, V, VI, VII, and VIII are absent. This study has included determination of limited types of serotype (i.e., Ia, Ib, and II to VIII). A similar study in Tehran, Iran, showed that serotype III was predominant (65.8%). Different studies performed in Japan revealed that serotypes VI and VIII were predominant among the Japanese population. Researchers showed the prevalence of serotype III could range from 29%–54% [23].

From Table 1, among 125 samples, 24 (19.2%) isolates were found to be GBS positive. Furthermore, using the PCR, 13 (54.17%) were found typeable GBS positive for four different serotypes, whereas 11 (45.83%) were nontypeable GBS. In another study performed by (Chaudhary M. et al.) in India (13%), China (20%), Brazil (28.4%), and the United States, 1%–2% of nontypeable isolates were found, respectively [20]. Possibly, vigorous capsular expression, necessary for identifying CPS by molecular methods, is less common in some regions of the world, including Nepal. GBS is serologically classified into nine types based on the CPS produced but it is introduced as heterogeneous serotype groups because of the variation found in genetic and protein levels. Molecular serotyping of GBS has been done by using multiplex PCR. However, the distribution of GBS serotypes has been also determined by using surface proteins, such as C alpha protein, C beta protein, Rib, and the R proteins (R1 through R4); these proteins are strongly connected with specific cps serotypes. For example, serotype Ia of GBS expresses C alpha protein, serotype III expresses Rib, and serotype V is associated with R1 and R4 proteins. GBS isolates which do not react with the standard cps antisera are defined as nontypeable (NT). About 2.9% and 1.4% GBS isolates in the United States are found as colonizing and invasive NT, respectively, and 12% of GBS isolates were found to be NT in Mexico. Previous studies characterizing GBS isolates have found a relationship between DNA macrorestriction band patterns and serotype/protein profile, especially for types V/R1, R4 and NT/R1, and R4 isolates. Another study found remarkable heterogeneity within the NT/R4 group compared with typeable isolates. However, the distribution of molecular serotypes and surface protein antigen genes associated with NT isolates is not known. Therefore, the basis of expressed surface proteins and by a variety of genetic typing methods more complete analysis of these NT have been done, which is not performed in this study [24]. In addition, considering PCR as a gold standard, low sensitivity of PCR was reported in this study which was consistent with PCR results obtained. The problem for this was maybe at sample preparation step and the cotton swabs used for sample collection contained charcoal in the transport media, which has been shown to lower the sensitivity of PCR. Even though other studies reported the sensitivity and specificity rate of PCR were 100% and 86.88%, respectively [8], it has been further revealed that the most invasive GBS infections (LOD) in infants, including all meningitis cases, are caused by serotype III. Available data reviewed by Sadowy et al. [25] epitomized that the prevalence of GBS serotype III could range from 29% to 54%, which is directly due to geographical area [23].

Table 2 shows that all 24 identified GBS isolates were tested against penicillin, ceftriaxone, levofloxacin, clindamycin, erythromycin, linezolid, tetracycline, ertapenem, chloramphenicol, and vancomycin. Antibiotics like tetracycline, levofloxacin, chloramphenicol, ertapenem, and linezolid were not recommended by CDC, but CLSI guideline and a previous research [26] included these antibiotics for antimicrobial susceptibility testing of *S. agalactiae*. We used these antibiotics for research purpose not for recommending the pregnant women. Among these antibiotics, ceftriaxone and linezolid were 100% effective against GBS followed by vancomycin, levofloxacin, and erythromycin. Penicillin, chloramphenicol, and clindamycin were effective to the GBS isolates but not 100% effective. GBS was resistant to tetracycline and ertapenem. As shown in Table 1, 50% of the GBS isolates were found susceptible to penicillin and 75% to vancomycin and 33.33% were resistant to erythromycin and 41.67% to clindamycin. This result is close to the one described by other authors, who have reported the sensitivity of penicillin and vancomycin being 57% and 76%, respectively. This observation may be attributed to the widespread and excessive use of antibiotics leading to rise in the antibiotic resistant organisms. Also the small number of samples may support the percentage rise in antibiotic resistant of GBS [27]. In addition, Moyo et al. [28], and Kimura et al. [29] reported reduced susceptibilities to penicillin and clindamycin. This result is not related to CDC and Brandon and Dowzicky [30] who found that group B streptococci (GBS) isolates were 100% susceptible to both penicillin and vancomycin. All the GBS isolates in this study were found resistant to tetracycline. In those with a high risk of anaphylaxis to penicillin and ampicillin, if the isolate is resistant to clindamycin and erythromycin, vancomycin is recommended [8].

In Table 2, 33.33%, 41.67%, and 100% GBS isolates were resistant to erythromycin, clindamycin, and tetracycline, respectively. Antibiotic resistance have been mediated by two principal mechanisms: methylation of ribosomal RNA, determined by erm genes, and active drug efflux by pumps encoded by mef genes [31] and tetracycline resistance is due to the effective transformation in plasmid [32]. Penicillin is the first choice drug, while ampicillin is an alternative, in cases of allergy to penicillin and at high risk for anaphylaxis; clindamycin and vancomycin are commonly recommended [27]. Due to the widespread use and even misuse of antibiotics in various clinical conditions, also their accepted efficacy of intrapartum prophylaxis in decreasing early-onset neonatal GBS infections has potentate the emergence of antibiotic resistance organisms. Antibiotic resistances among GBS were measured an increasing problem so that it was recommended to test the susceptibility of the other antibiotic as alternative choices for prophylaxis or treatment of GBS infection [8]. However, the Centers for Disease Control and Prevention privileges that the effectiveness of those different drugs, clindamycin, erythromycin, and vancomycin, as beta-lactam family has not been measured in controlled trials. Furthermore, the aptitude of these medicines to achieve antiseptic levels within the circulatory system and within the liquid body substance is extremely restricted [26].

Table 3 presents that, out of 24 PCR positive samples, the highest GBS rates were seen in age group 20–29 years, in 61–70 kg body mass, in formal education level ≤ Secondary Education Examination group, in case of parity both second and third pregnancy, and >36 weeks for gestational period, respectively, whereas the lowest GBS rates were seen in age group <19 years, 51–60 kg body mass, bachelor degree education, first pregnancy, and <36 weeks for gestational period, respectively. Statistically, there were no significant association found between age, body mass, formal education level, and parity with GBS as shown in table, while significant association was found between gestational period and GBS in this study. The pregnant women between 36^th^ week to the time of delivery may be highly prone to the GBS colonization.

In this study, the GBS rate was recorded slightly higher in medium aged women (see Table 3) than in other groups; a previous similar study reported higher rate in women with age 18–35 than in the younger [33]. In fact, sexual activity can be expected to be higher in medium age group as reproductive population which increases the risk of the GBS often being sexually transmitted [34].

According to the body mass, 61–70 kg weight group was found to exhibit the highest GBS rate (22.45%) followed by the weight groups 71< kg, 41–50 kg, and 51–60 kg (see Table 3). This may be due to the fact that the body mass is often related to the immune system of the body, therefore decreasing in immunity support the growth of the bacteria. Earlier studies have reported better risk of GBS maternal colonization with obesity and with increased maternal BMI [35]. In addition, the intestinal microbiota population differs in people with normal weight and with obesity [36–38]. Some studies have even identified to have increased GBS colonization among pregnant women with diabetes and obesity caused by other factors [39].

On the basis of formal education level (see Table 3), the highest GBS colonization rate was found for ≤ Secondary Education Examination (29.63%). Similar studies conducted by Monyama et al. [34] and Mengist et al. [40] showed higher GBS colonization in lower matrix. This may be due to the lack of awareness in uneducated or illiterate groups. Indeed, this study has unfolded the fact that the women with no formal education (29.63%) are more likely to be colonized with GBS. The relationship could be partly explained by the difference in personal hygiene, which is often better among educated population than the less educated one [41].

In case of parity, both second and third pregnancy showed higher GBS colonization rate; the colonization rate of GBS with respect to parity was higher in reproductive period of age and declined with the increasing age [42, 43]. The actual reasons for such variable colonization are unclear and need further study [44].

On the basis of gestational period, the highest GBS colonization rate (25%) was seen in >36 weeks period and the lowest GBS colonization rate (11.47%) was observed in <36 weeks period of pregnancy. Serotype III was found as dominant among the highest period group. Of all the factors examined, maternal colonization was significantly higher in women of gestation age >36 weeks than in women of gestation age between <36 weeks indicating an increased GBS carriage with gestation age [44]. The findings from this study might be unstable due to the small number of study subjects employed and time and budget limitations to find the possible risk factors. Thus, there is the need for a future large-scale study.

Screening pregnant women by rectovaginal swabs culture at 35 to 37 weeks of gestation is to improve the sensitivity and specificity of the identification of women who are colonized at the time of delivery [45]. A more rapid and sensitive method would be beneficial and cost-effective approach especially in developing countries [27]. Epidemiological surveillance of GBS is important to predict the spread of especial virulence clones [46]. Prophylaxis of neonatal GBS disease is currently based on screening of pregnant women during 35–37 weeks of pregnancy for colonization and then administering the recommended IAP [4].

There was no significant association between maternal ages, body mass, education, marital status, parity, and GBS. The small number of samples in this cohort and limited time and budget also may influence the result. However, gestational age has found a strong association with GBS. Vertical transmission of GBS to the newborn develops EOD [47]. These risk factors have not been studied in Nepalese pregnant women yet. The prevalence and distribution of GBS serotypes evaluated in this study will be valuable baseline data for the future study on distribution of GBS, studying invasive GBS serotypes, and the potential risk of neonatal GBS disease. Previously, in Nepal, reports on GBS serotype distributions and GBS antibiotic resistance have been rarely published.

## 5. Conclusions

All the isolates were sensitive to the antibiotics such as penicillin, erythromycin, chloramphenicol, clindamycin, vancomycin, linezolid, levofloxacin, and ceftriaxone, and were resistant to tetracycline and ertapenem. The current study demonstrated that type III was predominant and sensitive to all the drugs tested. The determination of prevalence of GBS would be significant for the future formulation of antibiotics which would form the basis for infection control.

The high colonization rate of GBS among pregnant women necessitates that the screening approach for all pregnant women at 35–37 weeks of gestation in all hospitals to provide antibiotic prophylaxis to GBS carrier must be recommended. Molecular typing of GBS is recommended to be performed to develop and implement effective prevention for pregnant women and neonatal GBS disease. In addition, it is an effective epidemiological tool for studying GBS. In this study, we observed resistance to clindamycin and erythromycin, which strongly supports the current CDC recommendation but linezolid and ceftriaxone were found 100% effective to GBS.

## 6. Limitations of the study

The present study included small sample size due to the limited study period and limited budgeting. This could equally be explained by the fact that the proportion of pregnant women met in the third trimester during the study period was small. Also due to lack of literature works, there was no possibility of comparing our results with that of others. The results were compared with the most important worldwide research concerning the prevalence of GBS. Since this study only focuses on the *S. agalactiae*, there can also be the case of vaginal candidiasis caused by certain fungi. This study included only nine serotypes out of 13 known virulent serotypes. Gene sequencing was not possible to perform in this study. Furthermore, the study does not distinguish between rural and urban prevalence and frequency of GBS colonization in women. This study focuses only on prevalence rate, and antibiotic resistance is also an important and valuable research which is not considered as the major part of the study due to lack of literature about GBS and their antibiotic resistance in Nepal.

## Figures and Tables

**Table 1:** Distribution of GBS in studied samples as well as typeable and nontypeable serotypes as observed in the GBS specimens.

Suspected samples size	GBS positive (%)	PCR results	
		Serotype positive (%)	Nontypeable (%)
125	24 (19.2%)	13 (54.25%)	11 (45.5%)

**Table 2:** Antimicrobial susceptibility pattern of GBS (*n* = 24) isolated from pregnant women.

Name of antibiotics (mcg)	Sensitive (%)	Intermediate (%)	Resistant (%)
Penicillin (5)	12 (50%)	—	12 (50%)
Erythromycin (15)	16 (66.67%)	—	8 (33.33%)
Chloramphenicol (30)	14 (58.3%)	2 (8.3%)	8 (33.33%)
Tetracycline (30)	—	—	24 (100%)
Vancomycin (30)	18 (75%)	—	6 (25%)
Ertapenem (10)	—	—	24 (100%)
Linezolid (30)	24 (100%)	—	—
Levofloxacin (30)	17 (70.8%)	3 (12.5%)	4 (16.7%)
Clindamycin (2)	14 (58.33%)	—	10 (41.67%)
Ceftriaxone (30)	24 (100%)	—	—

**Table 3:** Distribution of GBS and serotypes on the basis of age, body mass, formal education level, parity, and gestational period.

Age (years)	GBS positive (%)	Serotype positive				*p* value
		Ia	Ib	II	III	
<19 (*n* = 5)	1 (20%)	0	0	0	0	
20–29 (*n* = 101)	23 (22.77)	1	1	3	8	0.148
30< (*n* = 19)	0	0	0	0	0	
*p* value = 0.148, no association found between GBS and age of the pregnant women.						

Body mass (kg)						
41–50(*n* = 6)	1 (16.67%)	1	0	0	0	
51–60(*n* = 38)	6 (15.79%)	0	0	1	1	
61–70(*n* = 49)	11 (22.45%)	0	0	2	5	0.794
71<(*n* = 32)	6 (18.75%)	0	1	0	2	
*p* value = 0.794, no association found between GBS and body mass of the pregnant women.						

Formal education level						
≤secondary education examination (*n* = 54)	16 (29.63%)	1	0	3	4	
+2 (*n* = 42)	6 (14.28%)	0	1	0	2	0.285
Bachelor <(*n* = 29)	2 (6.89%)	0	0	0	2	
*p* value = 0.285, no association found between GBS and formal education level of the pregnant women.						

Parity						
First (*n* = 79)	14 (17.72%)	1	1	3	5	
Second (*n* = 36)	8 (22.22%)	0	0	0	2	0.893
Third (*n* = 10)	2 (20%)	0	0	0	1	
*p* value = 0.893, no association found between GBS and parity of the pregnant women.						

Gestational period						
<36 (*n* = 61)	7 (11.47%)	0	1	2	2	
>36 (*n* = 64)	16 (25%)	1	0	1	6	0.016
*p* value = 0.016, significant association was found between GBS and gestational period of pregnant women.						

## Data Availability

The data used to support the findings of this study are available from the corresponding author upon request.

## Abbreviations

CDC: Centers for Disease Control and Prevention
CLSI: Clinical Laboratory Standards Institute
CPS: Capsular polysaccharide
CTAB: Cetyltrimethylammonium bromide
DNA: Deoxyribonucleic acid
EOD: Early-onset group B streptococcal disease
GBS: Group B streptococci
IAP: Intrapartum antibiotic prophylaxis
LOD: Late Onset Group B Streptococcal Disease
MHA: Mueller–Hinton agar
PCR: Polymerase chain reaction
SEE: Secondary Education Examination
TE: Tris-ethylenediaminetetraacetic acid
UV: Ultraviolet.

## Supplementary Materials

Supplementary Materials Supplementary table: results from antimicrobial susceptibility test interpretive categories and Zone Diameter Breakpoint for *Streptococcus* spp. beta-hemolytic group, and GBS belongs to a beta-hemolytic group.
