# Supplementary material for: Molecular Characterization of Streptococcus agalactiae Isolates from Pregnant Women in Kathmandu City
Source: J Trop Med. 2020 Aug 28;2020:4046703. doi: 10.1155/2020/4046703 (PMC7474781; doi:10.1155/2020/4046703)
Supplement: Supplementary Materials — Supplementary table: results from antimicrobial susceptibility test interpretive categories and Zone Diameter Breakpoint for Streptococcus spp. beta-hemolytic group, and GBS belongs to a beta-hemolytic group. [file 4046703.f1.pdf]

Supplementary Table: Results from antimicrobial susceptibility test interpretive categories and Zone Diameter Breakpoint; for *streptococcus* spp.beta-Hemolytic Group and GBS belongs to a beta hemolytic group

Antibiotics type	Disk Content (mcg)	Interpretive categories and zone diameter breakpoint (nearest whole in mm)		
		Susceptible	Intermediate	Resistant
Penicillin	10	≥24	-	-
Erythromycin	15	≥21	16-20	≤15
Chloramphenicol	30	≥21	18-20	≤17
Tetracycline	30	≥23	19-22	≤18
Vancomycin	30	≥17	-	-
Ertapenem	10	≥24	-	-
Linezolid	30	≥21	-	-
Levofloxacin	5	≥17	14-16	≤13
Clindamycin	2	≥ 19	16-18	≤15
Ceftriaxone	30	≥24	-	-
